# Supplementary material for: Sex and rank in public service hierarchies: Rank distribution in Ghana’s health and security services
Source: PLoS One. 2026 Feb 9;21(2):e0342478. doi: 10.1371/journal.pone.0342478 (PMC12885314; doi:10.1371/journal.pone.0342478)
Supplement: Attachment — Submitted filename: RESPONSE TO EDITOR COMMENTS_13_01_2026.docx [file pone.0342478.s003.docx]

**RESPONSE TO EDITOR COMMENTS**

**Editor Comments:**

*The topic focuses narrowly on sex and rank distribution within the Ghana Police Service, whereas the abstract extends beyond this scope to include the Ghana Health Service, the Armed Forces, physical assessment tests, and the Affirmative Action Act (2024). This creates a misalignment between the title and the content described in the abstract. Kindly adjust either the topic or the abstract. A study of this nature should also have acknowledgement.*

**Response**

We appreciate this suggestion.

i. In response to the editor’s comment, the title has been revised to “Sex and rank in public service hierarchies: Rank distribution in Ghana’s health and security services” to align fully with the broader institutional scope described in the abstract.
ii. An acknowledgement statement has been added to the revised manuscript, in line with journal requirements, to appropriately recognise institutional support and ethical oversight.

**Journal Requirements:**

*If the reviewer comments include a recommendation to cite specific previously published works, please review and evaluate these publications to determine whether they are relevant and should be cited. There is no requirement to cite these works unless the editor has indicated otherwise. 

Please review your reference list to ensure that it is complete and correct. If you have cited papers that have been retracted, please include the rationale for doing so in the manuscript text, or remove these references and replace them with relevant current references. Any changes to the reference list should be mentioned in the rebuttal letter that accompanies your revised manuscript. If you need to cite a retracted article, indicate the article’s retracted status in the References list and also include a citation and full reference for the retraction notice.*

**Response**

We carefully reviewed the reference list to ensure completeness, accuracy, and consistency with the in-text citations, in line with PLOS ONE requirements. During this review, we identified three references that were included in the reference list but not cited in the main text, namely

1. Encyclopaedia Britannica. (n.d.). Brown v. Board of Education of Topeka. In Britannica.com. Retrieved August 24, 2024, from

https://www.britannica.com/event/Brown-v-Board-of-Education-of-Topeka

2. Loden, M. (1987). Recognising women's potential: No longer business as usual. Management review, 76(12), 44.
3. Norman, I. D. (2024). *Identity politics in Sub-Saharan Africa.* Cambridge Scholars Publishing. Lady Stephenson Library, Newcastle upon Tyne, NE6 2PA, UK.

These references have been removed from the reference list to ensure that all listed references are explicitly cited in the manuscript.

In addition, the reference list was checked for bibliographic accuracy and relevance. We confirm that no retracted articles are cited in the revised manuscript; therefore, no justification for citing retracted literature or inclusion of retraction notices was required. Any necessary adjustments to the reference list have been made accordingly and are reflected in the revised submission.
