# Supplementary material for: Sex and rank in public service hierarchies: Rank distribution in Ghana’s health and security services
Source: PLoS One. 2026 Feb 9;21(2):e0342478. doi: 10.1371/journal.pone.0342478 (PMC12885314; doi:10.1371/journal.pone.0342478)
Supplement: Attachment — Submitted filename: RESPONSE TO REVIEWER COMMENTS (2025PLOS).docx [file pone.0342478.s002.docx]

**RESPONSE TO EDITORIAL TEAM COMMENTS**

**Response to Comment 1**

Thank you for the feedback. I confirm that this study involved the analysis of fully anonymised secondary administrative data from the Ghana Police Service. No identifiable personal information (names, service numbers, contact details, or any traceable identifiers) was accessed by the research team at any stage.

As indicated in the Ethical Clearance Letter and Data Access Letter (both already uploaded to the system):

1. The Institute for Security, Disaster and Emergency Studies (ISDES) granted ethical approval for the study.
2. The data were provided by ISDES after they had conducted the review and anonymisation.
3. No medical records or patient samples were used.

**Response to Comment 2**

Thank you for alerting me. I have corrected the repository link in the Data Availability Statement. The dataset has now been re-uploaded. The updated link is: https://zenodo.org/records/17747718

**RESPONSE TO REVIEWER COMMENTS**

**Comment 1**

*Replace “available on request” with public deposition of an anonymized dataset sufficient to reproduce Tables 2, 5–7 and the OLR, along with SPSS syntax/output. If restrictions exist, specify the legal/ethical constraints, provide a minimal dataset, and indicate a data access mechanism consistent with PLOS policy.*

**Response**
Thank you for this important recommendation. An anonymised minimal dataset sufficient to reproduce all statistical analyses reported in this study (including Tables 2 and 5–7) has been deposited in a public repository (https://zenodo.org/records/17747718). The repository also contains the SPSS syntax file (analysis_syntax.sps) and the SPSS output file (analysis_output.spv) used to conduct the chi-square tests and the ordinal logistic regression

**Comment 2**
“The text references 55,129 GPS officers (as of March 2025) but the analytic sample is 43,968 valid records. Please reconcile: e.g., explain missingness, exclusions (invalid/duplicate/missing rank), or time windows.”

**Response**
Thank you for drawing attention to this inconsistency. The manuscript has now been clarified. The correct number of serving Ghana Police Service personnel as of March 2025 is **43,968**, comprising **12,945 females (30%)** and **31,023 males (70%)**. This represents the whole administrative population extracted from the Personnel Management Information System for the analysis. The figure of 55,129 does not apply to the period under study, and any possible ambiguity has now been removed from the text.

**Comment 3**
“Condense extended discussions of U.S. affirmative action jurisprudence; ensure precision (e.g., Bostock v. Clayton County concerns Title VII sex discrimination, not admissions-based affirmative action).”

**Response**
Thank you for this helpful observation. We have substantially condensed the discussion of U.S. affirmative action jurisprudence to maintain focus on the Ghanaian context. The revised text now briefly references key U.S. cases only to illustrate the general evolution of equality jurisprudence. We also corrected the earlier imprecision regarding Bostock v. Clayton County and now clarify that it concerns Title VII sex discrimination rather than admissions-based affirmative action. Longer quotations and extended legal background have been moved to the Supplementary Material (Section A1). These updates improve accuracy, conciseness, and alignment with the objectives of the paper.

**Comment 4**
*“Move lengthy quotations and some theoretical background to Supplementary Material, summarizing key points in the main text.”*

**Response**
Thank you for this valuable recommendation. We have now streamlined the main text by removing lengthy quotations and extended theoretical discussions. In the manuscript, we substituted concise summaries that preserve the key conceptual and legal arguments relevant to the study. This revision improves clarity, readability, and alignment with PLOS formatting expectations.

**Comment 5**
*“Standardize terminology and ensure consistent capitalization.”*

**Response**
Thank you for highlighting this issue. We conducted a comprehensive review of terminology, capitalization, and rank titles across the manuscript. Institutional names, rank designations, and statistical terms have now been standardized.

**Comment 6**
*“Ensure table labelling is self-contained.”*

**Response**
Thank you for this helpful observation. All tables have been revised to ensure that they are fully self-contained.

**Comment 7**
*“Proofread for redundancies and minor grammatical issues; trim long sentences for readability.”*

**Response**
Thank you for this comment. We have conducted a thorough proofreading of the manuscript and made multiple revisions to improve clarity and readability. Redundant phrases were removed, minor grammatical errors corrected, and several long or complex sentences were shortened. These changes enhance the overall flow of the paper without altering its substantive content.
